# The Effect of Patient-Related Factors on the Primary Fixation of PEEK and Titanium Tibial Components: A Population-Based FE Study

**DOI:** 10.3390/bioengineering11020116

**Published:** 2024-01-25

**Authors:** Corine E. Post, Thom Bitter, Adam Briscoe, Inger van Langen, René Fluit, Nico Verdonschot, Dennis Janssen

**Affiliations:** 1 Orthopaedic Research Laboratory, Radboud University Medical Center, 6525 GA Nijmegen, The Netherlands; 2 Invibio Ltd., Thornton Cleveleys FY5 4QD, Lancashire, UK; 3 Faculty of Science and Engineering, University of Groningen, 9747 AG Groningen, The Netherlands; 4 Laboratory for Biomechanical Engineering, Faculty of Engineering Technology, University of Twente, 7522 NB Enschede, The Netherlands

**Keywords:** total knee arthroplasty, polyetheretherketone, cementless tibial knee component, finite element simulation, micromotion, population study

## Abstract

Polyetheretherketone (PEEK) is of interest as implant material for cementless tibial total knee arthroplasty (TKA) components due to its potential advantages. One main advantage is that the stiffness of PEEK closely resembles the stiffness of bone, potentially avoiding peri-prosthetic stress-shielding. When introducing a new implant material for cementless TKA designs, it is essential to study its effect on the primary fixation. The primary fixation may be influenced by patient factors such as age, gender, and body mass index (BMI). Therefore, the research objectives of this finite element (FE) study were to investigate the effect of material (PEEK vs. titanium) and patient characteristics on the primary fixation (i.e., micromotions) of a cementless tibial tray component. A total of 296 FE models of 74 tibiae were created with either PEEK or titanium material properties, under gait and squat loading conditions. Overall, the PEEK models generated larger peak micromotions than the titanium models. Differences were seen in the micromotion distributions between the PEEK and titanium models for both the gait and squat models. The micromotions of all tibial models significantly increased with BMI, while gender and age did not influence micromotions.

## 1. Introduction

While titanium is currently the default material for cementless tibial total knee arthroplasty (TKA) components, polyetheretherketone (PEEK-OPTIMA^TM^) is of interest as an alternative implant material due to its potential advantages. First, PEEK can be used as implant material in patients with metal hypersensitivity [1]. Second, metal artefacts on MRI and CT images are avoided during the analysis of the peri-prosthetic tissue with a non-metal TKA [2]. Lastly, from a biomechanical perspective and with a potentially even larger effect, PEEK has a stiffness similar to human bone, which may contribute to avoiding peri-prosthetic stress-shielding [3,4]. However, this difference in stiffness between PEEK and titanium may also influence the primary fixation. 

The primary fixation of the cementless tibial component is essential for bone growth on and into the implant surface, which occurs during the first few weeks to months after the TKA procedure [5]. This primary fixation (before ingrowth has occurred) is governed by the compressive and shearing forces that are generated during the implantation of the press-fit components and are retained to some extent during dynamic loading. Apart from the frictional properties of the implant surface, the primary fixation depends on the design and material of the titanium component. When introducing a new implant material for TKA designs, it is therefore important to study its effect on the primary fixation.

The primary fixation can be evaluated by studying micromotions between the tibial bone and tray. A systematic review of Kohli et al. reported a micromotion threshold of 112 µm for osseointegration [6]. Such micromotions can be studied using computational tools, such as finite element (FE) analysis. Earlier FE studies on cementless PEEK tibial components focused on the analysis of a tibial model with parametric variations in the interface characteristics [7]. However, in clinical practice, the outcome may be influenced by patient factors such as age, gender, and body mass index (BMI). For instance, several studies have investigated the influence of BMI on the outcomes of TKA [8,9,10]. While there has been controversy as to whether or not a high BMI negatively affects the primary fixation, a recent study has shown that a high BMI causes larger micromotions in reconstructions with metal implants [9]. The influence of a high BMI on the primary fixation of a cementless PEEK tibial component, however, is currently unknown. We hypothesize that BMI may jeopardize the primary fixation of cementless tibial implants and that this effect may be more pronounced in cementless PEEK implants than cementless titanium implants. By adopting a population-based approach, we aimed to gain more insight into potential risk factors in patient populations. 

The research questions of this study were: (1) What is the effect of simulated implant material (PEEK vs. titanium) on tibial micromotions within a population? (2) Is the primary fixation of a cementless tibial tray influenced by patient characteristics (gender, age and BMI)? (3) Is there a difference in response as quantified by micromotions between PEEK and titanium material? 

## 2. Materials and Methods

### 2.1. CT Database

An anonymized CT database was created with ethical committee approval (reference number METC Oost-Nederland: 2021–13277). The patients included in this CT database were originally diagnosed with Kahler’s disease. This diagnosis was chosen as these patients undergo high resolution CT scans with a field of view that includes both knee joints. All CT scans were checked for not having pathologies in the knee joint. Additionally, gender, age, weight, and height were collected. In total, 74 tibiae (37 left) of 41 patients were included in the study (Table 1). 

### 2.2. Workflow FE Model

A total of 74 FE models were created of tibial TKA reconstructions with a tibial tray and a polyethylene insert. The models were analyzed with tibial trays with either PEEK or titanium material properties and were subjected to a gait and squat activity, resulting in 296 simulations in total. The models were created using an automated workflow, which is explained below in further detail.

#### 2.2.1. Segmentation

The left and right tibia of all patients were segmented from the CT scans using a convolutional neural network (https://grand-challenge.org/algorithms/tibia-segmentation-in-ct/) (accessed on 1 June 2022) [11]. The .mha segmentation files produced by the algorithm were subsequently converted into surface meshes (.stl files) using Matlab (Mathworks Inc. 2021b, Natick, MA, USA). 

#### 2.2.2. Orientation of the Tibia and Implant Alignment

An anatomical reference frame was assigned to the surface meshes of the tibia based on a previously defined coordinate system [12]. The tibiae were then aligned according to the orientation of the musculoskeletal model that was used to determine the implant-specific contact forces and centers of pressure of the loading conditions (see also “Boundary conditions, loads and contact interactions”). A coherent point drift registration was performed to find the rotation and translation for aligning the tibiae with the musculoskeletal model. CAD models of a generic cementless tibial tray and tibial insert were used. The size of the tibial tray and insert were chosen by inspecting the overlap of the contact area of the tibial tray with the cutting area of the proximal tibia. The proximal tibia was cut 8.9 mm distally from the articulating surface, such that the tibia, including tibial tray and tibial insert, was the same length as the length of the original tibia. The tibial tray size was chosen such that the bone surface was covered optimally with a maximum allowed overhang of 2 mm.

#### 2.2.3. Bone Cuts

The simulated bone cuts were made based on the distal surface of the tibial component using modeling software (HyperMesh 2020, Altair Engineering, Troy, MI, USA), achieving an ideal implant–bone interface. Additionally, the tibia was cut distally at 100 mm from the joint line.

#### 2.2.4. Volume Meshing

The surface meshes of the tibia, tibial tray, and tibial insert were converted into volume meshes consisting of tetrahedral elements (HyperMesh 2020, Altair Engineering, Troy, MI, USA). An edge length of 2.0–2.5 mm was used for the tibia, tibial tray, and tibial insert meshes, according to a previously performed mesh convergence study [13]. 

#### 2.2.5. Bone Material Properties Assignment

CT values were converted to bone mineral density values for each bone element using a calibration method that uses the known Hounsfield unit intensities for air, fat, and muscle [14]. Typical Young’s modulus values for trabecular and cortical bone are in the range of 5.0–20.5 GPa [15]. To allow for simulating permanent deformation of the bone during the virtual implantation, the bone was modeled as an elastic–plastic material using a Von Mises yield material model [16]. The tibial tray and tibial insert were modeled as an elastic isotropic material. The tibial tray was assigned with the material properties of either PEEK (3.7 GPa) or titanium (109 GPa). The tibial insert was modeled with the material properties for ultra-high molecular weight polyethylene (0.974 GPa) (Table 2).

#### 2.2.6. Boundary Conditions, Loads, and Contact Interactions

A single-sided touching algorithm was used to model the interaction between the tibial tray and the tibial bone. Friction between the tibial tray and tibia was modeled using a bilinear coulomb friction model. A coefficient of friction of 0.5 and an interference fit of 500 µm was applied through the contact algorithm on the whole implant surface that was in contact with the bone [17]. To obtain the desired interference fit of 500 µm, the interference fit was linearly increased during a virtual implantation phase. During the virtual implantation phase, typically, elastic and plastic deformations would occur in the bone, near the interface with the implant. The interference fit was kept constant at its maximum value during the loading phase. 

**Figure 1:**
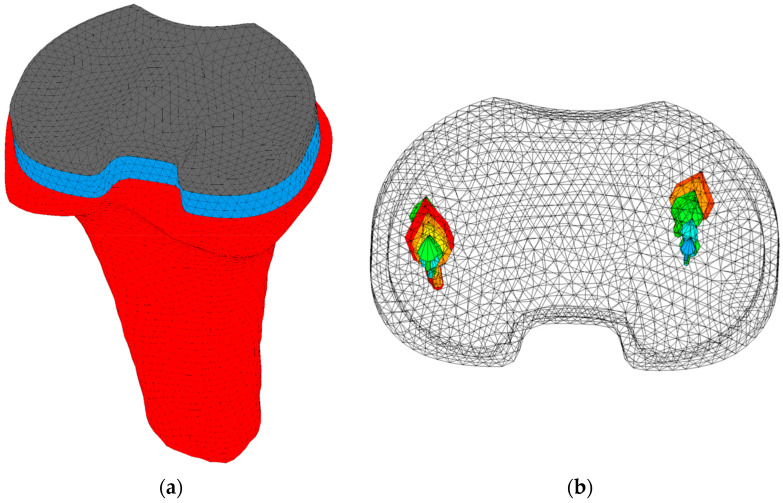
(**a**) Model of a right tibia (red), including tibial tray (blue) and tibial insert (grey). The model was fixated distally in all directions. (**b**) The medial and lateral tibiofemoral forces of a squat cycle are represented by the arrows. Larger arrows represent larger forces.

Implant-specific tibiofemoral contact forces and centers of pressure of gait and squat activities were derived from a musculoskeletal model that was modified by incorporating the current femoral and tibial implant design in the model [18]. The forces varied during the activities, with a maximum value of 1514 N. The centers of pressure were adapted according to the implant size. The contact forces were scaled based on the patient’s bodyweight and applied during four loading cycles to allow for the (numerical) settling of the implant. One loading cycle consisted of 73 increments for the gait activity and 67 increments for the squat activity. The tibia was fixated at the distal end in all directions. An example of a model, including tibia, tibial tray, and tibial insert, is shown in Figure 1.

### 2.3. Outcome Measures

The micromotions were defined as the relative displacements between the bone and the implant in the shearing direction using the same technique as Van der Ploeg et al. [19]. More specifically, the motions of the contact nodes on the implant surface were tracked relative to the contact faces of the bone surface. Each contact node on the implant surface was projected onto the closest contact face on the bone surface. The relative displacement of the contact node on the projected surface was calculated during the whole simulation. Regions with a large normal gap, for example, due to overhang, were excluded from the results. For each model, the resulting micromotions were defined as the largest distance during the full fourth loading cycle. The maximum resulting micromotion value was the maximum value of the contact nodes on the implant interface at a specific moment in time. The 95th percentile of the maximum resulting micromotions was taken to remove the nodes that potentially represented outliers. We analyzed the 95th percentile of the maximum resulting micromotions visually via the distributions on the interface of the tibial tray and quantitatively using violin plots. A violin plot shows the median, interquartile range, and outliers (similar to a boxplot), but it also visualizes the distribution of the data. 

### 2.4. Statistics

A linear mixed model was performed to evaluate the statistical significance of the interactions between micromotions and gender, age, and BMI of the patients included in the population. Micromotion was taken as the dependent variable, and the fixed effects included gender, age, and BMI. The analysis was conducted using IBM SPSS Statistics 27. The results were presented as regression coefficient (β) and its 95% confidence interval.

## 3. Results

**Figure 2:**
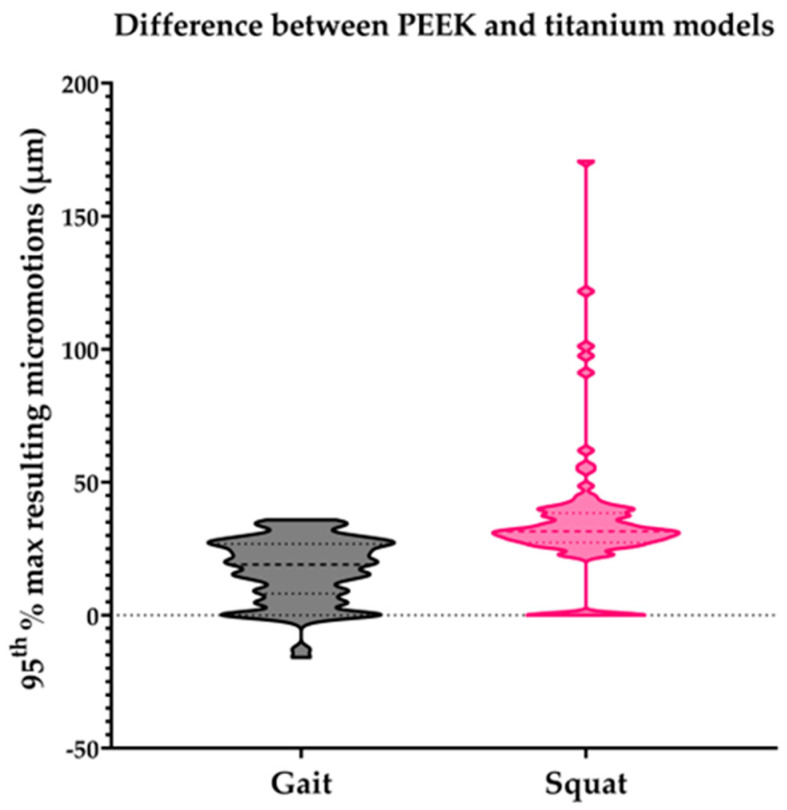
Difference in the 95th percentile of the maximum resulting micromotions between the PEEK and titanium models within all individual subjects of the population for the gait and squat models. Micromotion values for titanium models were subtracted from PEEK model values for each specific model.

Overall, the PEEK models generated larger peak micromotions than the titanium models (68 vs. 39 µm on average). For PEEK, the range of peak micromotions varied from 32.7 to 210.4 µm, while for titanium, a range of 6.8 to 90.3 µm was found. Under the squat load, the PEEK models generated larger peak micromotions than the titanium models, while under the gait load, some PEEK models generated slightly smaller peak micromotions than the titanium models (Figure 2). 

**Figure 3:**
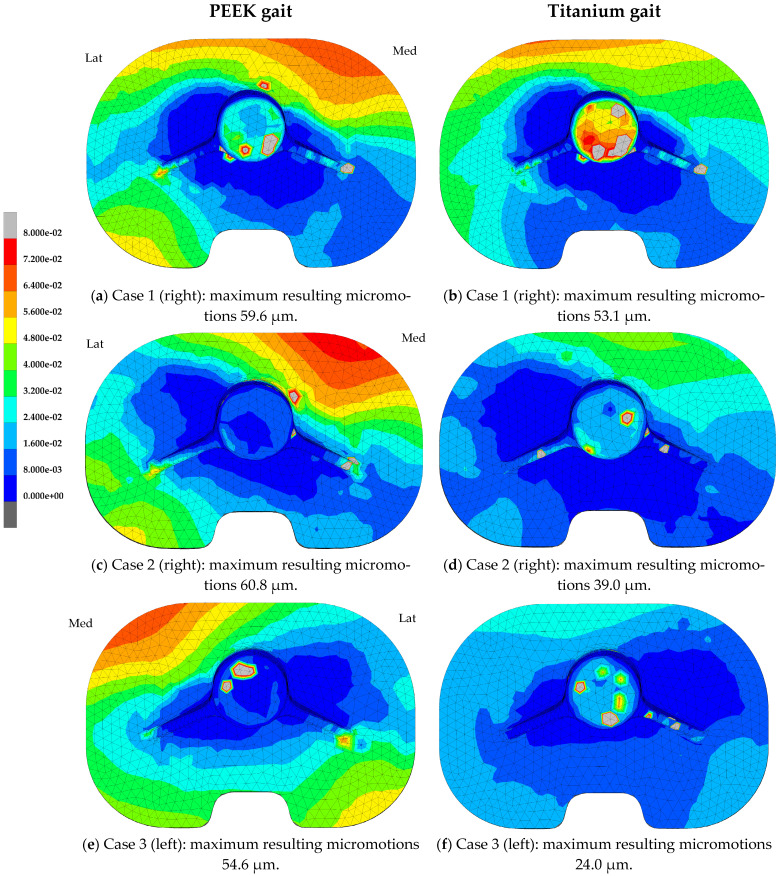

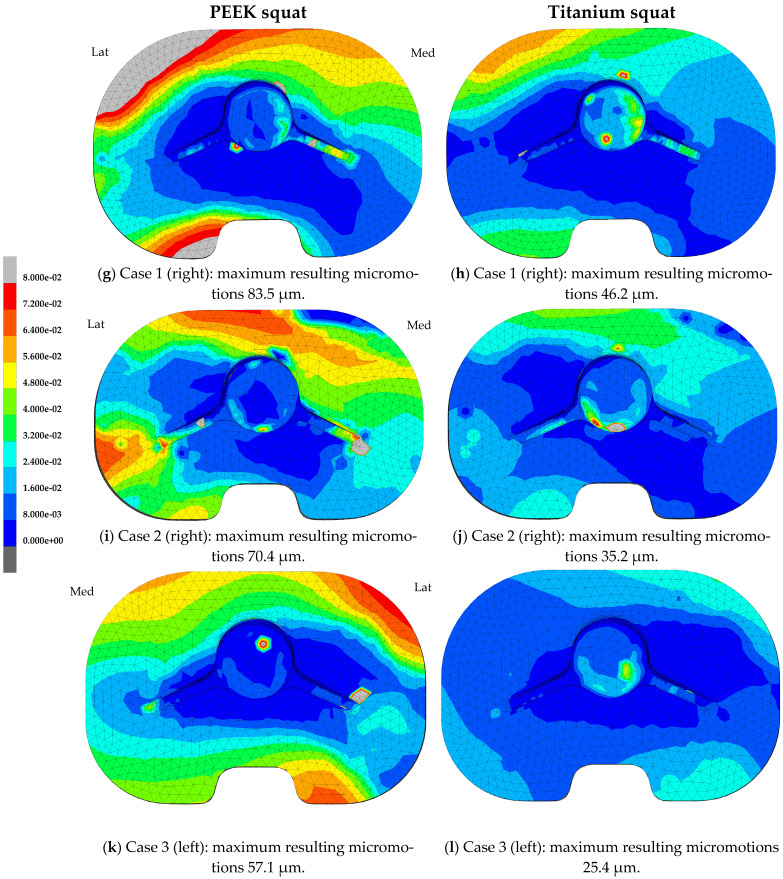
Resulting micromotion distribution (mm) at the implant interface of the tibial tray after the 4th loading cycle of the gait and squat activity. Case 1: Case with large maximum resulting micromotions within the population (man, right, 70 years, BMI 19.2 kg/m^2^). Case 2: Case with average maximum resulting micromotions within the population (woman, right, 62 years, BMI 31.2 kg/m^2^). Case 3: Case with small maximum resulting micromotions within the population (woman, left, 62 years, BMI 24.0 kg/m^2^).

Differences were seen in the distribution of the micromotions between the PEEK and titanium models for both the gait and squat models. Figure 3 shows the micromotion distributions for three specific cases within the population, with a PEEK and titanium tibial tray with relatively large (case 1), average (case 2), and small (case 3) maximum resulting micromotions, emphasizing the variations seen within this population of tibial models. For the titanium models, the largest resulting micromotions were concentrated around the anterior side of the tibial tray, during both the gait (Figure 3b,d,f) and squat activity (Figure 3h,j,l). The micromotions seen in the titanium models were comparable under squat and gait loads. In the PEEK models, the largest resulting micromotions under gait load were found at the anterior medial side and posterior lateral side (Figure 3a,c,e). During the squat activity, the largest resulting micromotions were concentrated on the lateral side both anteriorly and posteriorly (Figure 3g,i,k). 

**Figure 4:**
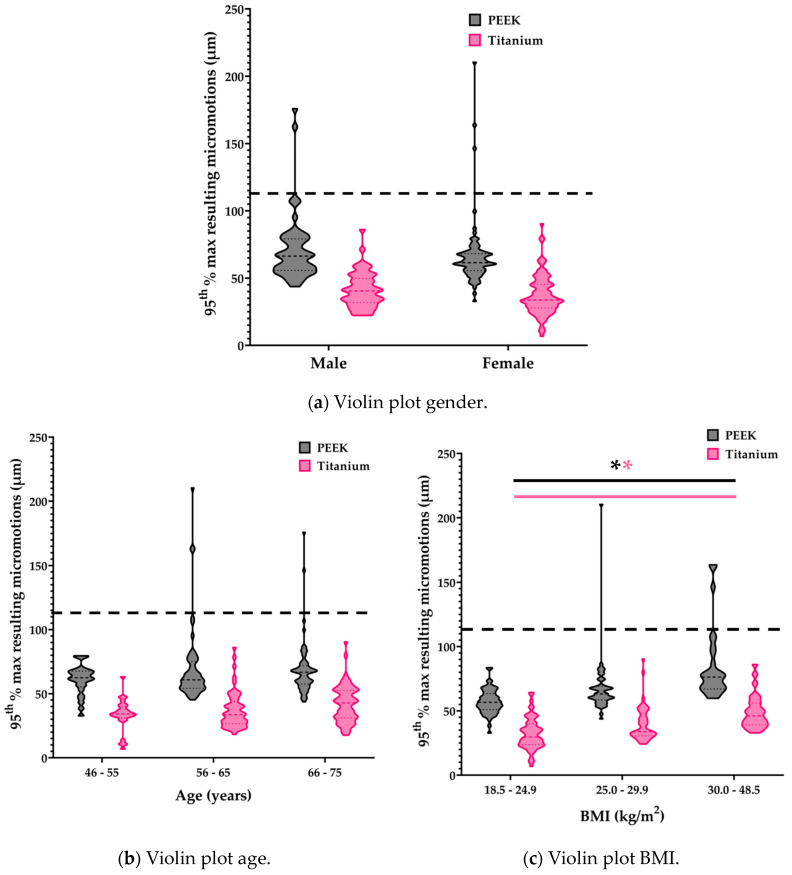
Violin plots of gender, age, and BMI. Statistically significant interactions are indicated with an asterisk. The dotted line represents the osseointegration threshold of 112 µm [6].

The micromotions of all tibial models significantly increased with BMI: 1.563 ± 0.254 µm (95% CI: 1.064, 2.063) (*p* < 0.001) (Figure 4). This was independent of the implant material. Neither gender (*p* = 0.639) nor age (*p* = 0.251) of the patients had a significant effect on the micromotions. 

## 4. Discussion

In this study, we assessed the effect of tibial tray material (PEEK vs. titanium) on tibial micromotions within a population and studied the influence of the patient characteristics gender, age, and BMI on these micromotions. The results of the current FE study indicate that, within the current cohort of models, peak tibial micromotions were larger for the tibial trays with PEEK material properties than for the tibial trays with titanium material properties. However, only relatively small differences were seen in the distributions of the peak micromotions on the tibial tray between the PEEK and titanium models. Additionally, the locations of the micromotions were similar among the cases included in this cohort, with generally higher micromotions on the anterolateral and medial side and the posterolateral side. The magnitude of the micromotions, however, were clearly different among the cases. In this cohort, only BMI had a significant influence on micromotions in both the PEEK and titanium models.

The overall current finding that PEEK material led to larger micromotions is not in agreement with an earlier study in which the effect of material properties (simulating either PEEK or titanium) of a cementless tibial component were compared with parametric variations of the interface characteristics [7]. In that study, no distinct differences were seen in the micromotion values and distributions between the PEEK and titanium models. In addition to the fact that only a limited number of cases was studied, this may be due to the fact that loads were applied in a different way (using Orthoload data with a single load application point vs. the application of more physiological loading conditions, including shifting centers of pressure in the current study). The application of implant-specific loading conditions that include the shift of the centers of pressure seems essential when investigating the primary fixation of a cementless PEEK tibial component. This is also in agreement with the study of Gonzalez et al., who also used implant-specific loading locations and found similar micromotion distributions to those in the current study, with the largest micromotions at the anterolateral side of the tibial tray [20].

The average maximum micromotion value in our previous study was around 40 µm for both the PEEK and titanium material, while in the current study, the average maximum micromotion was 68 µm and 39 µm for PEEK and titanium implants, respectively. In the current study, we also found gait models with larger micromotion values for the titanium component than the PEEK component, which emphasizes the importance of including a larger patient population with an inherently wider range of patient variations, to obtain more robust results. 

While the maximum resulting micromotions were higher in the PEEK models, the micromotions were below the threshold of 112 µm for bone ingrowth at the vast majority of the implant surface [6,21]. However, as indicated by the systematic review of Kohli et al., the threshold for osseointegration has been subject to debate. A threshold of 112 µm would, however, indicate that, also for a PEEK tibial component, a large portion of the implant–bone interface has favorable conditions for osseointegration and long-term implant fixation. Nonetheless, extending the current computational results to clinical practice is challenging. In the current study, we focused on the peak micromotions to assess the primary fixation, while there may be regions on the tibial tray surface that are more important for the primary fixation than others. Such knowledge, either from animal studies or clinical retrieval studies, may provide valuable additional information for the interpretation of the current results.

In the current study, only BMI had a significant effect on the micromotions, with a higher BMI leading to higher micromotions. In line with this result, Wan et al. concluded that a high BMI causes higher micromotions in both a gait and deep knee bend activity [9]. We also considered the effect of weight instead of BMI, which also was a statistically significant parameter in the linear mixed model. However, as BMI is a widely accepted parameter that also takes into account body composition, we chose BMI over bodyweight as a primary patient factor. Currently, not much is known of the influence of gender or age on the primary fixation of cementless tibial components. Gibbons et al. studied the risk of implant subsidence in elderly women who are more at risk for osteoporosis and found no increased risk in this patient population [22]. In our study, we did not find a correlation between elderly women and large micromotions, either. However, it is expected that the reduced bone quality influences the primary fixation, as good bone quality is a prerequisite. A larger population with a more in-depth analysis of BMD may be required to further elucidate this relation.

There are several limitations to the current study that may affect the interpretation of the results. As mentioned earlier, the first limitation refers to the bone quality of the models in this study. The analysis of a single parameter describing the overall bone quality of the models would have been useful for analyzing the effect of bone quality on micromotions. The second limitation relates to the number of cases included in this study. Although this is the first simulation study on the effect of patient characteristics on the primary fixation of cementless tibial components, a study including more cases is required for the analysis of the outliers, which may provide more detailed information on specific risk factors for patients receiving a TKA. Additionally, although we did incorporate the plasticity of the bone during implant insertion, we did not include viscoelastic behavior of the bone, while this may influence the compressive forces generated by the interference fit. Only a single implant alignment strategy was adopted, while variations in alignment may lead to differences in the loading configuration, possibly affecting micromotions at the implant–bone interface. Similarly, ligaments and muscles were not included in the current loading configuration. Another point refers to the implementation of BMI in the models by using a bodyweight correction of the applied forces. A larger bodyweight led to higher forces and therefore larger micromotions. However, a similar correction was not made for gender, age, or related changes in activity levels. Lastly, the models simulated in our study were generated with an idealized implant–bone interface, while cutting errors that occur in clinical practice may influence the primary fixation, leaving gaps at the interface and creating less optimal conditions for bone ingrowth. 

## 5. Conclusions

In conclusion, the current population study provides insights into the variation in primary fixation of a cementless tibial component in a cohort of computational models with varying patient characteristics. Although the distributions of the micromotions were comparable within this population, large differences in micromotion values were observed amongst the cases. PEEK models generated larger maximum micromotions than the titanium models, except for a few gait models. BMI was a significant affecting parameter in the primary fixation of cementless tibial components. 

Future research should focus on further elucidating the association between implant stiffness and primary fixation and whether there is an optimal stiffness that provides a good balance between primary fixation and long-term effects, such as stress shielding. In addition, a more in-depth multivariate analysis is required to highlight the effects of the interactions of patient characteristics, including bone quality, bone geometry, bone density, and implant fixation. Finally, an outlier analysis of a larger population may provide more insights into potential risk factors in patient characteristics for the primary fixation of cementless tibial components. 

## Figures and Tables

**Table 1:** Patient characteristics (n = 41).

Gender, n (%)	
M	15 (37%)
F	26 (63%)
**Age** in years, mean (range)	63 (46–75)
**Height** in m, mean (range)	1.71 (1.55–1.99)
**Weight** in kg, mean (range)	79 (52–170)
**BMI** in kg/m^2^, mean (range)	26 (18–49)

**Table 2:** Material properties FE model.

Component	Material	Stiffness (GPa)
Tibia	Bone	5.0–20.5
Tibial tray	PEEK	3.7
	Titanium	109
Tibial insert	Ultra-high molecular weight polyethylene	0.974

## Data Availability

Data are contained within the article.

## References

[1] Innocenti M., Vieri B., Melani T., Paoli T. (2017). Metal hypersensitivity after knee arthroplasty: Fact or fiction?. Acta Biomed..

[2] Nishio F., Morita K., Doi K., Kato M., Abekura H., Yamaoka H., Kakimoto N., Tsuga K. (2023). Radiopaque properties of polyetheretherketone crown at laboratory study. J. Oral Biosci..

[3] De Ruiter L., Janssen D., Briscoe A., Verdonschot N. (2017). A preclinical numerical assessment of a polyetheretherketone femoral component in total knee arthroplasty during gait. J. Exp. Orthop..

[4] Rankin K.E., Dickinson A.S., Briscoe A., Browne M. (2016). Does a PEEK Femoral TKA Implant Preserve Intact Femoral Surface Strains Compared With CoCr? A Preliminary Laboratory Study. Clin. Orthop. Relat. Res..

[5] Hofmann A.A., Bloebaum R.D., Bachus K.N. (1997). Progression of human bone ingrowth into porous-coated implants. Rate of bone ingrowth in humans. Acta Orthop. Scand..

[6] Kohli N., Stoddart J.C., van Arkel R.J. (2021). The limit of tolerable micromotion for implant osseointegration: A systematic review. Sci. Rep..

[7] Post C.E., Bitter T., Briscoe A., Verdonschot N., Janssen D. (2023). The sensitivity of the micromotions of a cementless PEEK tibial component to the interface characteristics. SSRN.

[8] Başdelioğlu K. (2020). Effects of body mass index on outcomes of total knee arthroplasty. Eur. J. Orthop. Surg. Traumatol..

[9] Wan Q., Zhang A., Liu Y., Chen H., Zhang J., Xue H., Han Q., Wang J. (2023). The influence of body weight index on initial stability of uncemented femoral knee protheses: A finite element study. Heliyon.

[10] Daniilidis K., Yao D., Gosheger G., Berssen C., Budny T., Dieckmann R., Holl S. (2016). Does BMI influence clinical outcomes after total knee arthroplasty?. Technol. Health Care.

[11] Sendak M.P., Gao M., Brajer N., Balu S. (2020). Presenting machine learning model information to clinical end users with model facts labels. NPJ Digit. Med..

[12] Miranda D.L., Rainbow M.J., Leventhal E.L., Crisco J.J., Fleming B.C. (2010). Automatic determination of anatomical coordinate systems for three-dimensional bone models of the isolated human knee. J. Biomech..

[13] Berahmani S., Janssen D., Wolfson D., de Waal Malefijt M., Fitzpatrick C.K., Rullkoetter P.J., Verdonschot N. (2016). FE analysis of the effects of simplifications in experimental testing on micromotions of uncemented femoral knee implants. J. Orthop. Res..

[14] Eggermont F., Verdonschot N., van der Linden Y., Tanck E. (2019). Calibration with or without phantom for fracture risk prediction in cancer patients with femoral bone metastases using CT-based finite element models. PLoS ONE.

[15] Keyak J.H., Falkinstein Y. (2003). Comparison of in situ and in vitro CT scan-based finite element model predictions of proximal femoral fracture load. Med. Eng. Phys..

[16] Keyak J.H., Kaneko T.S., Tehranzadeh J., Skinner H.B. (2005). Predicting proximal femoral strength using structural engineering models. Clin. Orthop. Relat. Res..

[17] Post C.E., Bitter T., Briscoe A., Verdonschot N., Janssen D. (2022). A FE study on the effect of interference fit and coefficient of friction on the micromotions and interface gaps of a cementless PEEK femoral component. J. Biomech..

[18] Marra M.A., Andersen M.S., Damsgaard M., Koopman B.F.J.M., Janssen D., Verdonschot N. (2017). Evaluation of a Surrogate Contact Model in Force-Dependent Kinematic Simulations of Total Knee Replacement. J. Biomech. Eng..

[19] Van der Ploeg B., Tarala M., Homminga J., Janssen D., Buma P., Verdonschot N. (2012). Toward a more realistic prediction of peri-prosthetic micromotions. J. Orthop. Res..

[20] Quevedo Gonzalez F.J., Lipman J.D., Lo D., De Martino I., Sculco P.K., Sculco T.P., Catani F., Wright T.M. (2019). Mechanical performance of cementless total knee replacements: It is not all about the maximum loads. J. Orthop. Res..

[21] Engh C.A., O’Connor D., Jasty M., McGovern T.F., Bobyn D., Harris W.H. (1992). Quantification of Implant Micromotion, Strain Shielding, and Bone Resorption With Porous-Coated Anatomic Medullary Locking Femoral Prostheses. Clin. Orthop. Relat. Res..

[22] Gibbons J.P., Cassidy R.S., Bryce L., Napier R.J., Bloch B.V., Beverland D.E. (2023). Is Cementless Total Knee Arthroplasty Safe in Women Over 75 Y of Age?. J. Arthroplast..

